# The Effects of CD73 on Gastrointestinal Cancer Progression and Treatment

**DOI:** 10.1155/2022/4330329

**Published:** 2022-05-17

**Authors:** Pengcheng Sun, Xiao Zheng, Xiaodong Li

**Affiliations:** ^1^Department of Oncology, The Third Affiliated Hospital of Soochow University, Changzhou, China; ^2^Department of Tumor Biological Treatment, The Third Affiliated Hospital of Soochow University, Changzhou, China; ^3^Jiangsu Engineering Research Center for Tumor Immunotherapy, Changzhou, China; ^4^Institute for Cell Therapy of Soochow University, Changzhou, China

## Abstract

Gastrointestinal (GI) cancer is a common and deadly malignant tumor. CD73, a cell-surface protein, acts as a switch of the adenosine-related signaling pathway that can cause significant immunosuppression. Recent evidence has emerged that CD73 is a promising immunotherapy target for regaining immune cell function and restraining tumorigenesis, and a growing stream of research indicates that combining immunotherapy with other therapies can effectively improve the prognosis and survival of GI cancer patients. Several immune checkpoint inhibitors have been approved for use in GI cancer recently; however, they have demonstrated limited efficacy. Solving the problem of immunosuppression in GI cancer is the key to developing an effective therapeutic option and the modulation of CD73 expression may provide an answer. In this review, we discuss current research on CD73 in gastric, liver, pancreatic, and colorectal cancer to evaluate its therapeutic potential as an immunotherapy target in GI cancers.

## 1. Introduction

Gastrointestinal (GI) cancer is one of the most common and malignant cancers worldwide, accounting for 21% of new cancer cases in 2020. Presently, the mainline therapeutic options for GI cancers, namely surgery, radio/chemoradiotherapy, and targeted therapy, have demonstrated limited efficacy [1]. With our improved understanding and progress of cancer immunotherapy, including the anti-PD-1/PD-L1 and anti-CTLA4 monoclonal antibody (mAb)-based therapies as well as the expansion of immunotherapeutic clinical trials on various types of cancers, the prospect of developing an effective treatment for GI cancers has been enhanced [2]. However, immunotherapy is still in its infancy due to the complexity and diversity of the tumor-immune system, and the limited number of approved drugs suitable for personalized cancer treatment make GI cancer management challenging. Therefore, it is necessary and urgent to study other molecular pathways to explore other novel therapeutics.

CD73, encoded by *NT5E* (5′-Nucleotidase Ecto) gene, is a ubiquitous cell-surface protein in humans. As the switch molecule of the adenosine-related signaling pathway, CD73 can hydrolyze extracellular adenosine monophosphate (AMP) to adenosine and regulate various biological and cellular activities [3]. In the tumor microenvironment (TME), CD73 is significantly overexpressed on the surface of both tumor cells and non-tumor cells. It has recently been shown that CD73's expression is abnormally up-regulated in a wide spectrum of cancers, including gastric cancer (GC), colorectal cancer (CRC), breast cancer (BC), and hepatocellular carcinoma (HCC) [4, 5]. The elevated expression of CD73 is implicated in the enhanced ability of cancer cells to proliferate, invade, and metastasize, resulting in a poor prognosis [6]. CD73-derived adenosine can inhibit the function of protective immune cells such as CD8^+^ T cells and NK cells and simultaneously increase the number of immunosuppressive cells like regulatory T cells (Tregs) and myeloid-derived suppressor cells (MDSCs) by binding to adenosine receptors, thus promoting the immune escape mechanism [7, 8]. Recent studies indicate that CD73 can be activated by HIF-*α*, IFN, and Wnt-mediated signaling and regulated by miRNA as well [9–12]. In response to complement-approved anti-PD-1/L1 and anti-CTLA-4 antibody therapies, the suppression of CD73 can enhance the therapeutic activity of monoclonal antibodies and repress immunotolerance of cancer cells [13]. Clinical trials of anti-CD73 mAbs underline the potential of CD73 as a molecular target to improve the efficacy of immunotherapy, as well as prognosis and survival in GI cancer patients [14]. This article reviews the significance and mechanism of CD73 in the major GI tumors, as well as its potential as a therapeutic target combined with existing treatment options.

## 2. The Structure and Functions of CD73 in TME

CD73 is a cell-surface protein anchored by a glycosylphosphatidylinositol (GPI) to the cell membrane. It consists of 548 amino acids with a molecular weight of 70 kDa [15]. Structurally, CD73 represents a dimeric form whose N-terminal domain coordinates the binding of metal ions, and the C-terminal domain acts as the binding pocket for AMP [16]. Generally, CD73 plays a role in immune regulation, inflammatory tissue homeostasis, and pathophysiological responses [17]. It is normally expressed in various tissues and organs, as well as in leukocytes and endothelial cells, and has regulatory roles in the modulation of inflammatory factors such as IFN-*α*, IFN-*β*, and lipopolysaccharide (LPS) [18]. However, CD73 is significantly overexpressed in many cancers and is partly related to the expression of hypoxia-inducible factor-1 (HIF-1) in the tumor microenvironment. HIF-1 contains *α* and *β* subunits, of which the *α* subunit determines the activity of HIF-1 [19]. HIF-1*α* is widely overexpressed in multiple tumors and can enhance the expression of CD73 [9], but the specific induction mechanism is still unclear.

**Figure 1:**
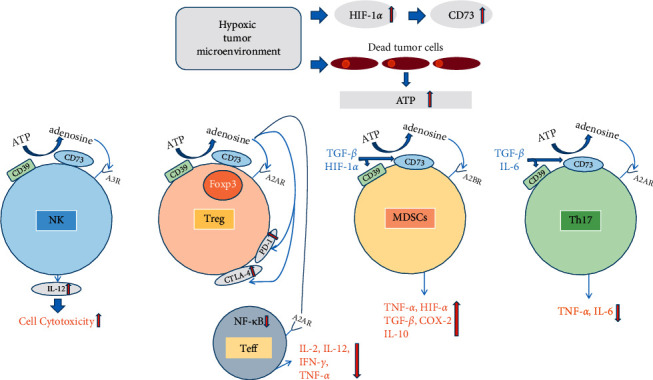
CD73 influencing immune cell expression in the tumor microenvironment. Hypoxic TME upregulates the expression of HIF-1*α* and CD73 and causes more ATP release from dead tumor cells. Through the CD39 and CD73 on immune cells, ATP is hydrolyzed to adenosine. On NK cells, adenosine activates A3R and causes the upregulation of IL-12 which enhances the cytotoxicity of NK cells. On Foxp3+Treg cells, adenosine activates A2AR and increases the expression of immunosuppression checkpoints such as PD-1 and CTLA-4. Adenosine can also activate A2AR on T effector cells and downregulate the expression of NF-*κ*B in T effector cells, and then decrease the production of cytokines such as TNF-*α* and IFN-*γ*. On MDS cells, adenosine can activate A2BR and increase the production of cytokines such as TNF-*α*, HIF-*α*, and TGF-*β* while some of these cytokines in turn can regulate the expression of CD39 and CD73 on MDS cells and Th17 cells. On Th17 cells, adenosine can activate A2AR and decrease the production of cytokines such as IL-6 which also in turn can regulate the expression of CD39 and CD73 on Th17 cells.

The tumor-promoting effect of CD73 in the TME can be mediated through both non-enzymatic and enzymatic pathways. In the non-enzymatic pathways, CD73 regulates the interaction between cells and extracellular matrix (ECM) components by acting as a signaling adhesion molecule and thereby promoting the adhesion and metastasis of cancer cells [20]. In the enzymatic pathways, CD73 coordinates with CD39 to hydrolyze the damaged cell-secreted ATP to adenosine and phosphate [17, 21]. Adenosine exerts its effect by activating four widely distributed G protein-coupled receptors A1R, A2aR, A2bR, and A3R [22]. Physiologically, A1R can regulate adenylate cyclase (AC), calcium channels, potassium channels, and phospholipase C (PLC) through Gi protein coupling. A2AR and A2BR are coupled to Gs/Golf protein and Gs/Gq protein, respectively, and enhance the activity of AC, cyclic AMP, PLC, and protein kinase (PKC). A3AR can inhibit AC and stimulate PLC activity through Gi/Gq protein coupling [23]. However, all of them can activate the mitogen-activated protein kinase (MAPK) signaling pathway and the phosphorylation of extracellular signal-regulated kinase (ERK)1/2 [24] split and treat as new paragraph. The co-existence of CD39, CD73, and adenosine receptors on multiple immune cells has become a prerequisite for immunomodulation. When CD73-derived adenosine activates these receptors, the functions of immune cells are restricted with the concomitant increase in the expression of immunosuppressive molecules such as PD-1/PD-L1 [25]. On Foxp3^+^ Treg cells, CD39 co-expressed with CD73 to produce adenosine which can stimulate Treg cells' proliferation and inhibit the activation of Nuclear Factor-Kappa Beta (NF-*κ*B) in Teff cells via A2aR, thereby reducing the release of a broad range of proinflammatory cytokines and chemokines, further enhancing the immunosuppressive activities of Treg cells [26, 27]. On CD4^+^T cells and CD8^+^T cells, CD73-derived adenosine can inhibit the proliferation and differentiation of these T cells through A2aR and A2bR [17, 28]. CD73-derived adenosine can also reduce the secretion of IL-2, TNF, and IFN-*γ* from CD8^+^ T cells and upregulate the expression of immune checkpoints such as lymphocyte-activation gene 3 (LAG-3) and PD-1 [29]. On the surface of T helper 17 (Th17) cells, CD73 is co-expressed with CD39 to generate adenosine, but this activity is tightly regulated by interleukin-6 (IL-6) and transforming growth factor-*β* (TGF-*β*) [30]. On macrophages, CD73-derived adenosine can suppress the generation of IL-12, NO and macrophage inflammatory protein-1*α* (MIP-1*α*). Conversely, CD73-derived adenosine can increase the generation of IL-10 by reducing the production of TNF-*α* via A2aR and A2bR [31]. Furthermore, CD73-derived adenosine can also enhance IL-4- and IL-13-induced alternative macrophage activation through A2bR [32]. Adenosine generated by CD73 on natural killer (NK) cells also enhances the cytotoxic immune response of NK cells by A3R activating the activity of IL-12 [33]. In the hypoxic tumor microenvironment, the immunosuppressive effect is enhanced due to the increase of adenosine caused by more CD73 expression and ATP release. Moreover, tumor infiltrating immune cells like MDS cells can establish autocrine and paracrine adenosine signaling loops to persistently upregulate CD73 and activate A2bR. These signaling pathways can also induce the expression of HIF-1*α*, TNF-*α*, IL-10, TGF-*β*, and other factors to inhibit the anti-tumor immune response [34, 35] (Figure 1).

Furthermore, CD73 can affect the AKT/GSK-3*β*/*β*-catenin signaling pathway through A2aR by inducing the cyclinD1 activation to alter the cell cycle status of cancer cells and promote tumorigenesis [36]. Additionally, CD73 can also act on A2aR, A2bR, and A3R to increase the production and release of vascular endothelial growth factor (VEGF) through adenosine generated by the enzymatic pathway, as well as promote the movement of microvascular endothelial cells [37, 38]. On the other hand, CD73 promotes the formation of tubular structures through the non-enzymatic pathway, thus contributing to angiogenesis [39]. In conclusion, CD73 plays an essential role in promoting the occurrence, development, and immune escape of tumor cells through various immunomodulatory pathways.

## 3. CD73 in Gastric Cancer

Gastric cancer (GC) ranks fourth in mortality around the world, and early-stage surgery is the only effective treatment. Since 2017, anti-PD-1/PD-L1 mAb immunotherapy has become an important option for the treatment of GC [40]. However, immunosuppression makes such treatment effective only for a small subset of patients [41]. Hence, further studies are needed to find broad-spectrum small-molecule targets to improve the efficiency of immunotherapy in GC.

As a powerful immunosuppressant, CD73 modulates tumor survival and progression. It has been shown that compared with normal gastric tissues, GC tissue contains a higher level of CD73 protein which is closely related to the stronger invasiveness, short overall survival (OS) and disease-free survival (DFS), advanced clinical stage, deeper tumor invasion, and distant metastasis via immunohistochemistry, the Kaplan-Meier analysis, univariate Cox proportional hazards modeling, and fluorescent staining [42–44]. In contrast to paracancerous tissue, the expression of CD73 is stronger in the intra-tumoral tissue [44]. The gene set enrichment analysis (GSEA) revealed that most CD8^+^T cells in CD73-overexpressing tumors are exhausted by functionality, and the remaining cells are dysfunctional with decreased secretion of interferon-gamma (IFN-*γ*), granzyme B (GZMB), perforin, and CD107a, but having an increased expression of PD-1. This study also indicates the overactivation of multiple tumor-promoting pathways, including the epithelial-mesenchymal transition (EMT) and hypoxia signaling pathway [44]. All of these could be relevant to the development of immune-escaping TME and the poor efficacy.

Reinhardt and his team discovered that c-Jun, a transcription factor that is significantly co-expressed with CD73 mRNA, can induce the upregulation of CD73. Furthermore, in the intronic enhancer region of the CD73 gene, they found a critical c-Jun binding site that controls the c-Jun/AP-1-dependent transcriptional activation and inflammatory cytokine signaling downstream of the mitotic MAPK signaling pathway in melanoma [45]. In GC, c-Jun can also enhance the transcription of CD73 and upregulate its expression to promote metastasis [43].

GTPase-activating protein RICS is highly expressed in CD73-overexpressing GC cells, according to the GenBank and gene transcription results. It is known that RICS can increase the expression of *β*-catenin, vimentin, and Snail proteins and decrease the level of cadherin by inhibiting the activity of its direct substrate RhoA [46]. Through immunoblotting assay, Xu and his team found that while inhibiting RhoA, RICS could block the phosphorylation of RhoA's direct substrates LIMK and cofilin to enhance the EMT process. They also have demonstrated that the activity of RICS can be suppressed by inhibiting CD73-derived adenosine and restored by both selective and non-selective adenosine receptor agonists [43]. These results further confirmed that CD73 could promote the EMT mechanism by regulating the RICS/LIMK/cofilin signaling axis via adenosine receptors.

Tumor cells usually exhibit high glycolytic properties due to the hypoxic and low pH conditions inside the TME, which is called the Warburg effect [47]. Cao and his team identified that CD73 expression is associated with the Warburg effect in the differentially expressed genes (DEGs) related to the glycolysis in GC cells. They found that enhanced expression of CD73 can significantly increase the expression of genes associated with glucose uptake, lactate production, and glycolysis (*GLUT1*, *HK2*, *ENO1*, *LDHA*), indicating the involvement of the Warburg effect [48]. The accelerated Warburg effect eventually leads to rapid tumor cell proliferation, drug resistance, and stemness induction in GC cells. Therefore, CD73 is a tumor-promoting molecule that affects tumor progression in multiple pathways and can be a promising target for GC treatment.

## 4. CD73 in Hepatocellular Carcinoma

Hepatocellular carcinoma (HCC) ranks third in mortality around the world, and the 5-year overall survival rate after radical resection is only 25–30% [49]. Current immunotherapy is beneficial to only a minority of patients due to the complexity of the TME. Therefore, it is essential to investigate the pathological role of other key pathways to improve the therapeutic efficacy of immunotherapy.

CD73 is a recently discovered promising target involved in the tumor immune escape. Through immunohistochemical staining and data analysis, researchers have shown that the expression of CD73 is commonly slightly higher in the liver during viral hepatitis and alcoholic liver disease with cirrhosis conditions, but more abnormally higher in HCC [50]. The clinical data show that abnormal CD73 expression positively correlates with lymph node metastasis, poor tumor differentiation, and short OS [50, 51]. *In vitro* studies have exhibited that CD73 plays a crucial role in tumor suppression when it is expressed at the normal level but promotes HCC cell proliferation and metastasis with the concomitant development of EMT when overexpressed. Ma et al. reported that CD73 can raise the level of phosphorylated AKT and GSK-3*β* proteins in the PI3K/AKT signaling pathway through the binding of CD73-derived adenosine to A2aR, thus activating RAP1 and inducing PIP3 generation [51].

CD73 is also closely related to *α*-SMA^+^ tumor-associated fibroblasts (CAF). CD73 expression is higher in HCC with increasing numbers of *α*-SMA^+^ CAF, and CD73^+^ tumor cells mostly located at the CAF interface [52]. The reason for this could be the secretion of hepatocyte growth factor (HGF) by CAF at the interface. HGF can affect c-MET, which acts as a ligand of HGF [53], and then activate the MEK/ERK signaling to upregulate the expression of CD73 and promote the proliferation and metastasis of HCC cells [52].

Additionally, CD73 has a certain influence on liver cancer stem cells (CSCs) and the high expression level of CD73 protein is closely related to the formation of stem cell spheroids [54]. SOX9, as one of several stem cell-related genes, can be transcriptionally induced by c-Myc and phosphorylated by GSK-3*β*, which are key downstream targets of AKT signaling [55]. Since CD73 activates the PI3K/AKT signaling pathway, the expression of SOX9 can be stabilized with the help of CD73 to maintain the properties of CSCs [54]. Above all, CD73 has an undeniable promoting effect on a variety of cells in HCC through manifold pathways and has the potential to be one of the therapeutic targets of HCC.

## 5. CD73 in Pancreatic Cancer

Pancreatic cancer (PAC) has an extremely poor prognosis and caused nearly 370,000 deaths in 2020. The 5-year overall survival rate (OS) is still less than 25% after surgical resection combined with gemcitabine and paclitaxel. Combined immunotherapy can only prolong survival by up to one year [56]. Thus, it is very important to explore more molecular pathways of pancreatic cancer to find novel therapeutic drugs.

As a HIF-1*α* target gene, CD73, as well as TGF-*β*, IFN-*α*, and WNT, is significantly upregulated by hypoxia in the TME. The histological features of PAC are excessive connective tissue hyperplasia and decreased blood vessels, which contribute to a hypoxic TME [57]. Zhou et al. have demonstrated that the positive expression rate of CD73 in PAC is as high as 30.7%, and the protein is mainly located on the cell membrane and cytoplasm [58]. Chen et al. have determined that the expression of CD73 in PAC tissue is significantly higher than that in the normal pancreatic and adjacent tissues and positively correlates with the OS, tumor stage, and size. Moreover, they have found that the expression level of CD73 is negatively correlated with the DNA methylation level of the *CD73* gene in PAC [59]. This suggests that the abnormal expression of CD73 may have connections to its gene methylation level.

Tumor necrosis factor-*α* (TNF-*α*) is a pro-inflammatory cytokine produced by macrophages and can specifically bind to tumor necrosis factor receptor 1 (TNFr1), which is primarily responsible for apoptosis, as well as to tumor necrosis factor receptor 2 (TNFr2) that can induce the activation of the AKT/ERK signaling pathway [60]. Through the GSEA, Zhou et al. have revealed that the expression of *TNFr2* and genes of the tumor necrosis factor-*α* (TNF-*α*) signaling pathway are significantly changed with the expression of CD73. Upregulation of TNFr2 in CD73-overexpressing cells remarkably promotes the phosphorylation of AKT and ERK, while this effect is attenuated in CD73-depleted cells [58].

CyclinD1, as a downstream molecule of the AKT and MAPK signaling pathways, is an important protein that can promote the transition of cells from the G1 to the S phase during the cell cycle [36]. Researchers discovered that the expression of cyclinD1 along with the level of p-AKT and p-ERK was dramatically decreased when the expression of CD73 was suppressed [58]. This suggests that CD73 may promote cell proliferation by activating the AKT/ERK signaling pathways. However, the internal mechanism of the activation needs more research. In conclusion, CD73 may interfere with the activation of the AKT/MAPK signaling pathway and increase the expression of cyclinD1 in the cell cycle by modulating the expression of the *TNDR2* gene, thus playing an important role in tumor proliferation and metastasis.

## 6. CD73 in Colorectal Cancer

Colorectal cancer (CRC) is the third most malignant tumor in the world, and about 80–90% of patients use palliative chemoradiotherapy with limited effects to prolong their lifespans due to unresectable distant metastases [61]. The invention of targeted anti-PD-1/PD-L1 and anti-CTLA4 mAbs therapeutic options has improved the survival, but the differential nature of the TME limits the universality of the treatment [62]. Therefore, targeting other vital molecules in the TME is urgently necessary.

CD73 is found significantly upregulated in CRC tissues compared with normal tissues, and its high expression is closely related to poor tumor differentiation, extensive immune cell infiltration, short OS, advanced tumor stage, and metastasis according to the Kaplan-Meier analysis, univariate Cox proportional hazards regression model analysis, ROC curve, and multivariate analysis [63]. *In vitro* experiments have proved that tumor cells' colony formation and survival abilities are enhanced when the expression of CD73 is upregulated, along with the remarkably increased proportion of S phase cells. *In vivo* studies have demonstrated that the tumorigenic ability of CD73-overexpressing CRC cells is also dramatically stronger than that of CD73-downregulated cells [64].

CAF in CRC is also important for high levels of CD73 expressing cells. Yu et al. have identified that CD73 protein is highly concentrated in CAF in clinical CRC samples, and CD73's expression level is positively correlated with the CAF abundance in tumor cells and tumor-infiltrating lymphocytes (TIL) [65]. Through RNA sequence analysis, it has been found that A2aR and A2BR of adenosine are the major receptors expressed in CAF, and the expression of A2aR is much higher than that of A2BR [65]. Together, these findings indicate that CD73 in CRC is also a tumor-promoting molecule and is expected to become a treatment target soon.

## 7. The Potential of ANTI-CD73 Therapy in GI Cancers

The limitations of current therapeutic options for GI cancers are undermined by the immune escape mechanism. The importance of CD73-adenosine axis in GI cancers promotes further investigation to develop new drugs and targeting CD73 or adenosine receptors in combination with other treatments may improve the efficacy of immunotherapy and other non-surgical treatments.

Several studies have shown that the decrease in the proliferative and invasive abilities of most tumor cells, including GC, HCC, and BC, as well as the increased functions of immune cells such as CD8^+^T and NK cells can be observed after using the CD73's enzyme activity inhibitor APCP or anti-CD73 mAb [66, 67]. Tumor-specific VEGF level is significantly decreased as a result of these inhibitors, resulting in a significant reduction in tumor angiogenesis [68]. Because CD73-derived adenosine can activate widely distributed adenosine receptors which also play important roles in tumor immunosuppression, metastasis, and angiogenesis, inhibiting CD73 and adenosine receptors at the same time has a better anti-tumor effect [69]. This strategy was proved by Young and his team in the model that combined targeting of A2aR and CD73 in the treatment of lung tumor metastasis. They found that the involvement of NK and CD8^+^ T cells, and IFN-*γ* and Fc receptors is required for the optimal therapeutic activity of anti-CD73 and anti-A2aR mAbs [70]. Presently, a clinical trial involving the combination of AZD4635 (A2aR inhibitor) and MEDI9447(CD73 inhibitor) is being evaluated in the treatment of non-small cell lung cancer [71].

HIF-1*α* is associated with CD73 expression. Farnaz et al. used nanocarriers to introduce small interfering RNAs (siRNAs) into hypoxic tumor cells to simultaneously silence HIF-1*α* and CD73 molecules and found that the colony formation ability of tumor cells, compared with targeting HIF-1*α* or CD73 alone, was significantly inhibited and the expressions of VEGF, TGF-*β*, and fibroblast growth factor (FGF) were also significantly reduced. The tumor also shrunk evidently as expected [72].

In anti-PD-1/PD-L1 and anti-CTLA-4 mAb therapies, inhibiting the expression of CD73 and adenosine receptors can also enhance the therapeutic efficacy. Allard et al. have shown that anti-CD73 mAb can enhance the therapeutic activities of both anti-PD-1/PD-L1 and anti-CTLA-4 mAbs. The combination of anti-CD73 and anti-PD-L1 mAbs is more effective than the combination of anti-CD73 and anti-CTLA-4 mAbs for subcutaneous tumors and metastases [13]. Anti-PD-1/PD-L1 mAb can increase the expression level of A2aR [73], and the activation of A2aR enhances the expression of PD-1 on tumor-specific CD8^+^ T cells and CD4^+^ Foxp3^+^ Treg cells. However, anti-CD73 and anti-PD-1/PD-L1 mAb need the existence of IFN-*γ* and CD8^+^T cells to exert their anti-tumor effects [13]. This indicates that the combination of anti-PD-1/PD-L1 and anti-CD73 mAbs or A2aR antagonist can be more effective in exerting the synergistic effect of anti-tumor T cell-mediated immunotherapy. In addition, anti-PD-1/PD-L1 mAb can significantly enhance the antitumor activities of CAR-T cells and CAR-NK cells when combined with targeted A2aR or anti-CD73 mAb regimen [74, 75].

CD73 expression is associated with the tumor resistance to various drugs, such as Adriamycin [76]. In HCC, CD73^+^ tumor cells are highly resistant to the first-line targeted drug Lenvatinib [54], and patients with low CD73 expression in GC respond better to Pembrolizumab [44]. There are also related reports of drug resistance for other chemotherapeutic drugs, such as Carboplatin, Gemcitabine, and Paclitaxel [77].

Currently, clinical inhibitors targeting CD73 or A2aR are being tested in early clinical trials, including MEDI9447, BMS-986179, NZV930, and CPI006 [14, 71, 78]. Recent data show that MEDI9447 combined with Durvalumab has better safety and the results of the trial are relatively more satisfactory than other trials using different combinations [14]. Preliminary results from a Phase I/IIA study of BMS-986179 in combination with Nivolumab in advanced solid tumors have exhibited a similar safety profile to Nivolumab monotherapy [78]. In conclusion, although the current research suggests that anti-CD73 or anti-adenosine receptors can enhance the efficacy of other tumor treatment methods, the specific mechanism and the efficacy and safety of new drugs still need to be studied.

## 8. Conclusion

In-depth research in recent years has improved our understanding of CD73's mechanistic role from an inflammatory regulatory molecule to a key immunosuppressive and tumor-promoting molecule. The selective weakening of the function of CD8^+^ T cells, NK cells, and other immune cells by adenosine derived from the CD73 enzymatic pathway and activation of adenosine receptors provides strong evidence for the immune escape model. CD73 plays an important role in promoting GI tumorigenesis by interfering with the RICS/LIMK/cofilin signaling axis in GC, TNDR2 in PC, and RAP1 that activates the AKT and MAPK signaling pathways and effect the downstream molecules such as cyclinD1 and c-Myc in HCC. CD73 not only affects tumor cells but also has an important and stable effect on cancer stem cells and CAF. Both *in vitro* and *in vivo* experiments have demonstrated that the inhibition of CD73 and adenosine receptors can effectively block the tumor progression and has a good enhancement effect on the efficacy of other drugs such as anti-PD-1/PD-L1 antibodies. Relevant clinical drug experiments are also being carried out in a step-by-step manner. Cumulatively, these results have proved CD73's role in tumorigenesis in GI cancer and provided a new direction for relieving tumor immunosuppression and inhibiting tumor progression. However, specific mechanisms of action on signaling molecules or receptors such as RICS and AKT and whether CD73 affects the same molecule through the same pathway in different GI cancers are not well understood. At the same time, the upstream regulatory molecules of CD73 are not very precise and still need to be studied. Combination efficacy, toxicity, drug side effects, and the mechanism of action of the anti-CD73 mAb and anti-adenosine receptor preparation in combination with anti-PD-1/PD-L1 mAb, chemotherapy, and targeted therapy in GI cancers also need further experiments and research. However, the potential role of CD73 therapeutics is clearly important for the treatment of GI cancers. We believe that more research on CD73 in the tumor system will bring more hope and help for the treatment of cancers not limited to GI cancers.
